# Selections and Abstracts

**Published:** 1902-06

**Authors:** 


### *SELECTIONS AND ABSTRACTS.*

---

### Why not Forestall Tuberculosis and Blot It from the Face of the Earth? *

Modern sanitary laws enforced by municipalities and nationalities have partially throttled certain contagious and infectious diseases and increased longevity 16 per cent. within the last generation.

Vaccination was practiced more than one hundred years ago, but smallpox was not rigidly quarantined until later, and now it is to be observed that through vaccination and intelligent quarantine the disease is so robbed of its horrors, that it no longer strikes terror to a community.

By rigid quarantine of diphtheria and prompt administration of diphtheritic antitoxin, this once dreaded disease has become a subject of vigilance and not of fear.

The investigations of the cause of yellow fever by Walter Reed, of the United States Army at Havana, during the past two years have been so thorough and convincing of the one real source of infection, that never again will the United States or any other progressive country, with a well organized board of health, experience an epidemic.

Scientific investigation has set the trap and scattered the poison for the rat with a strict quarantine of every suspected case of the plague and has forever prohibited the disease again establishing a hold in any quarter.

By intelligent quarantine of scarlet fever, whooping-cough and measles, followed by thorough formalin fumigation, these diseases are limited to circumscribed localities, yes limited to a single person in a single room in a building with many rooms, or dormitories, with many persons of a susceptible age.

In the year 1899, approximate mortuary statistics of the United

---

*Read by Jas. A. Burroughs, M.D., Asheville, N. C., before the Tri-State Medical Society of the Carolinas and Virginia, at Asheville meeting, February, 1902.

States of America was 1,900,000. Pulmonary tuberculosis was responsible for 246,000 deaths, or approximately 13 per cent. of all deaths were due to pulmonary tuberculosis. The mortuary statistics of the United States possibly strike a fair average with other countries, except that of Germany, where in the same year 25 per cent. of all deaths in that country were attributed to tuberculosis. Insurance statistics of companies issuing policies against sickness in Germany for the year 1899, show 42 per cent. of the laboring class tubercular. These facts or statistics are so stupendous and startling that the average mind fails to grasp the cold, thin, clammy situation of this unfortunate multitude.

We should pause and think; practically no man is born tubercular; every one who has tuberculosis has contracted the disease and, in every instance, it could have been forestalled and prevented. For just twenty years we have known the tubercle bacillus, and year by year we have learned its habits and various modes of entering the human system, consuming and extinguishing the life of that which is made in God's own image.

No problem before us at present compares in gravity and magnitude, not only to this generation, but to those who come after us, as to the wisest disposal of the consumptive. The great question is of twofold significance: First, How can we best care for the patient; second, How can we prevent the spread of the disease?

How can we best care for a consumptive? To place him in the most favorable condition for an arrest or cure and, at the same time, prevent a spread of infection, allows of much latitude for debate, but at best, narrows down to a few cold facts. Education of the laity, suitable legislation and a hearty co-operation of each medical man in the land.

Tuberculosis annually causes more deaths than any other two diseases combined. It is an insidious disease, unlike acute infectious troubles, and the public has learned to look upon consumption as something that has always been with us, and accepts the situation as a matter that cannot be prevented.

How to educate the masses out of this state of lethargy is a question that should be begun under the head of hygiene in our public schools. Small children should be taught that tuberculosis is contagious so as to avoid infection from an afflicted parent, teacher or schoolmate. These children should be taught that in the sputum lies millions of germs, ready to infect any subject with a poor resisting power caused by heredity, malnutrition or any other cause.

Public schools everywhere should have a course on public hygiene, with special reference to obliterating tuberculosis. This course should include the poor resisting power of children born of tubercular parents, or born of healthy parents with systems that have deteriorated, because of environments, lack of proper food, clothing or habits.

Too much attention cannot be given to modern sanitary living quarters, with adequate room for an abundance of pure, fresh air and sunshine; at the same time devitalized air should be taught as one of the potent causes of tuberculosis. There are many things to be included in this little course of hygiene or young war against tuberculosis which has received no attention here but may be brought out in the discussion.

I know of no other way to reach the masses on this or any other subject of such vital importance, as starting at the fountain head with the little ones who in half a generation will have the custodianship of all nations.

We must all acknowledge that the contagion or infection of any disease must be implanted in the minds of a community before a moral support is given a health board to stamp out a disease. It is when boards of health have good laws to prevent the spread of tuberculosis, and these boards of health are "scotched" by the medical profession, upheld by a people who have been taught the nature of tuberculosis with its various modes of infection, etc., and the means by which it can be controlled, that we expect to see the disease practically abolished from mortuary certificates.

The public mind of America, as well as that of Europe, is becoming aroused as to the ravages of tuberculosis and, at the same time, as to its prevention and curability. Much unwise legislation has been proposed from erratic, impulsive sources, for instance, the "Immigrant Exclusion Act of the Tubercular." Some

States have introduced bills to prevent the introduction of the tubercular; even a few villages have passed ordinances prohibiting the tubercular from entering their gates. Such legislation is worthless, because it is impracticable, and will ever remain dead upon the statutes. Notwithstanding some mistakes have been made along this line, much good has already been accomplished. The civilized world is enlightened as to the great danger that lurks in the consumptive's sputum. (Various pocket flasks and hand cuspidors have been devised as receptacles for sputum; minute details are given each individual as to collecting and destroying same by all intelligent physicians.)

Four-fifths of the cities of the United States have passed ordinances prohibiting spitting on the sidewalks, floors of street-cars and public buildings. Asheville, N. C., in 1894, was the first town in the United States to pass such an ordinance. Dr. Trudeau, at Saranac Lake, very shortly followed suit, and it soon became contagious. Many hundred copies of our ordinance have been sent to city attorneys throughout the country, by request. The ordinance has been well observed here. No man can visit our principal cities to-day and compare hotel lobbies, street-car floors and sidewalks with six or eight years ago without being impressed with a change for the better.

Municipalities are discussing, on every hand, the disposition of their consumptive poor; in many instances homes have been established exclusively for the tubercular. Massachusetts and New York have already made contributions and established State institutions for consumptives; other States are formulating plans along the same line; it is entirely logical and but reasonable to expect every city and State in the Union to adopt some practical means for the care of the consumptive pauper and to prevent him from infecting others.

The Sanatoria idea has taken possession of Italy, Germany, and, I may say, all of Europe. As yet the Sanatoria capacity is so out of proportion to the number of consumptives in those countries that it offers but slight protection; besides, it has yet to be proven by any kind of statistics that this is best for the inmates. At best, this plan can only offer protection in ratio to the Sanatoria capacity.

The tenement house question has been well discussed by Knopf and others, and, in a few instances, has brought about some legislation as to capacity and ventilation of apartments. Many cities have taken up the milk and meat supply with a view to eliminating tubercular food products. Many States and nationalities have laws providing for disposal of tubercular stock. Many other legislative steps have already been taken against this, the great foe of the human race.

An International Congress of Tuberculosis has been formed and it is from this international body that the world may expect, in a reasonable time, some formulated plans whereby this disease may be controlled.

An international prophylaxis of tuberculosis is eminently necessary, and suitable international regulations or laws must and will be enacted to combat this disease, as has been with cholera, the plague and yellow fever.

In the meantime, while we are waiting for action of our International Congress to formulate laws, let each nationality, State, city and parish meet the question squarely with suitable care for this class. In this country it is a debatable question as to national, State, municipal or county supervision. Very few States possess suitable climatic points for this class. National consumptives' parks, consisting of small farms and truck gardens with many tents and inexpensive cottages, located in a dry climate at a suitable altitude, seems to be the reasonable solution. In this way the chief focuses of infection will be removed from the country and placed in a most advantageous position for a recovery. At the same time it is to be noted that sputum, in a high, dry, sunny climate is practically sterile as soon as dry, so these parks would not become infectious or pestiferous localities from long habitation.

In this connection I cannot refrain from suggesting something along the same line for the prospective consumptives; poor children born of tubercular parents with bad lymphatic systems and reared in an atmosphere loaded with moisture and superabundance of bacteria, and who have improper, scant food, are certain to become tubercular as time rolls on unless environments are altered. It is for this class, who are very numerous, that so much good may be accomplished by a change of environments and by giving them good food and plenty of fresh air.

A national supervision would be most admirable, but is probably impracticable; a country supervision is entirely feasible. It is the poor that needs government protection; the well-to-do and rich have largely ceased to raise tubercular offspring. The trouble is forestalled by the child being placed in the best climate with good food, etc., to grow up into a healthy citizen.

The medical world is aroused upon the subject, as is evidenced by a continuous stream of literature from all quarters of the globe.

Something has already been accomplished in lessening the number of deaths in the country. The mortuary certificates of five of our largest cities for 1900 show a decrease of 36 per cent. from tuberculosis over 1890; this intimates that the trouble is not only being prevented to a degree but is being handled in a more intelligent manner than ever.

With a course of public schools on hygiene with special reference to tuberculosis, in fifteen years we will have a voting population who will indorse any suggestion from the International Congress of Tuberculosis. This is a young subject, yet vigorous; some of the best brains of the world are devoting their energies to the prevention of the trouble.

While waiting for the educated hoard, international, national and State laws, let each physician be true to his clinician by keeping the young from becoming tubercular by knowledge which is in the possession of all; at the same time placing those who are already tubercular in a suitable climate in competent hands and fumigating behind them.

If we can and do utilize the knowledge that is in our possession, we will be able to largely forestall tuberculosis and so reduce the death-rate from this cause that longevity may be increased several per cent.—*N. C. Medical Journal.*

---

### TREATMENT OF PAIN IN THE EAR.

In *Treatment* for January, 1902, Yearsley, in an exhaustive article on this topic, says that the treatment of pain resulting from acute otitis media, whether of the catarrhal or purulent type, must be both local and general. The treatment of both types must be upon the same principle. The methods may be divided into three groups—prophylactic, abortive and operative. Of these the first need not be entered upon, as the author refers to cases of pain—that is, where the condition is already established, and therefore only considers the abortive and operative methods.

When an attack of acute middle-ear inflammation is established, prompt treatment is necessary. The matter of first importance, and one which should be secured at once, is absolute rest. The patient should be confined to his room, and if there is a likelihood of the case being of the suppurative type, he should be kept in bed. The ear must be protected from anything which is likely to increase the inflammation, and stimulating foods, alcoholic liquors, mental excitement, and physical exertion must be absolutely forbidden. A smart saline purge should be at once administered, and it will often be found useful to start with five grains of calomel, followed up in the usual manner with a Seidlitz powder.

Insomnia must be combated by means of an opiate, but the best method of procuring sleep is by allaying the pain. This can best be done by the application of heat or cold, or by the combination of the two already alluded to. As a general rule, heat is preferred by the patient. It may be employed dry by means of the hot-water bag, hot flannels, etc., or by moist instillation. In using hot instillations a temperature of 115° will suffice.

The continued influence of heat, however, keeps the ear in a condition of congestion which retards resolution and favors the continuance of inflammation, whilst the application of cold (especially to the parts surrounding the ear) often rapidly lessens the inflammation. Hartmann, therefore, suggested the combined action of the two agents, and claimed much benefit therefrom. The region below the auricle is covered with cold compresses or an ice-bag, and warm fluids are instilled into the meatus or hot sponges inserted. The author always employs this method, and finds it most useful.

But the best method of all, and the one which he most frequently uses, is the application of leeches. From two to four or six, applied just in front of the tragus, will often cut short the pain and inflammation with rapidity. Their application should be followed by dry heat by means of hot flannel or wool.

Otologists are divided in their opinion as to the wisdom of inflation during acute inflammatory conditions of the middle ear. To use the Politzer air-douche when the symptoms are at their height is to undoubtedly aggravate the condition; but when the inflammation is subsiding, the air-douche may be gently and cerefully used, the amount of pressure exerted being regulated and slight. Thus judiciously applied it will be found that patients are more quickly restored to hearing, and that the ventilation and consequent proper drainage of the tympanum accelerates the resolution of the inflammatory process.

In the early stages of acute inflammation of the middle ear the process can be aborted by the careful use of the Eustachian catheter. The following is a case illustrating this: On last Christmas Day a colleague of the writer came to him suffering from acute catarrhal inflammation of the tympanum. He had been in great pain all night, and his membranum tympani was much injected and bulging considerably in its posterior half, so much so as to quite obscure the handle of the malleus. After carefully cleansing the nares, the Eustachian catheter was passed and greatly inflated. One hour later the bulging was considerably less, and from that time recovery was rapid.

In a certain number of cases the treatment described will be found sufficient to abort altogether the attack, especially if the case be one of the catarrhal type. But, unfortunately, a certain percentage will require further measures. It is exceedingly unwise to wait too long and to continue to employ abortive treatment when it makes no impression. If the symptoms continue unabated for twelve hours, or if, whilst they diminish under treatment during the day, they return with their former or with increased severity during the night, then it is imperative that something further should be done. The middle ear is in a condition of extreme tension and must be relieved, and this brings us to the operative treatment. This consists in making an incision into the membrana tympani, an operation which has been called

"paracentesis," but which is better named simply "incision" or "myringotomy." By incising the drumhead much damage to the ear may be prevented, and since wounds in that membrane heal so quickly, it is far better to incise it unnecessarily than not at all. If the symptoms do not abate under abortive treatment, the incision of the membrane is imperative, whether the condition be one of simple or purulent inflammation. Prompt operation can do no harm, while its neglect may mean acute mastoid complications or chronic trouble, with all its attendant worries and drawbacks. If, on inspection, the membrane can be seen to be bulging, there is no reason for delaying incision, and when the surgeon has any doubt as to the propriety of the proceeding, he will find it the best and wisest course to incise.

As regards the *modus operandi*, there is not much to be said. Many special instruments have been devised, but the best is a Graefe cataract knife, or a small sharp tenotome. This should be as sharp as possible, since the keener the edge the less the pain of incision.

The meatus should be rendered as antiseptic as possible by swabbing with perchloride of Lister's strong mixture. If possible the patient should have gas, as the pain, though momentary, is extreme. The head requires to be carefully steadied, and the incision should be made where there is the greatest amount of bulging, and under the best possible illumination. If the membrane be bulging uniformly, the cut should be made in the posterior inferior quadrant. It should be bold and free, a mere puncture being useless. After incision, all that is required is a dressing of antiseptic gauze. Politzerization as a means of blowing secretion or pus out of the opening should not be practiced, as by blowing micro-organisms from the nasopharynx into the tympanum a simple case may be converted into a suppurative one; or, if the case be already purulent, inflation may drive pus into the attic or mastoid antrum. Nor is syringing or irrigation necessary, the gauze acting as an efficient drain. It is only when the contents of the tympanum are very tenacious that syringing is required.—*Ther. Gazette.*

### MODERN METHODS OF VACCINATION AND THEIR SCIENTIFIC BASIS.

In the London *Lancet* of December 21, 1901, Copeman writes an important paper on this subject, which is so interesting at the present time.

The treatment of the arm at the time of vaccination and subsequently during the progress of the case is another subject which has aroused considerable controversy, and concerning which much divergence of opinion would appear to exist. Thus in some quarters the initial cleansing of the arm is said to be objected to by the parents as a reflection on the care, or want of care, on their part as regards the condition of their children, but in general it is found that a little tactfulness in explaining the difference between ordinary and surgical cleanliness has sufficed to overcome the difficulty. In addition to this aspect of the case the friction employed in the process is of value in causing a slight capillary dilatation which undoubtedly contributes to the success of the operation. Water, soap and water, spirits of wine, or antiseptic solutions of greater or less potency containing boric or carbolic acids, lysol or perchloride of mercury, for instance, are employed by different operators for the purpose. Of these in all probability a warm solution of boric acid is the most generally useful, a stronger antiseptic, such as corrosive sublimate, unless removed by the subsequent use of sterilized water or alcohol, being liable to exert a somewhat deleterious effect upon the lymph.

The method to be employed at the operation and during the maturation of the vesicles for the protection of the vaccinated area from extraneous infection has not been defined by the regulations, for the reason that it appeared probable that each man would best attain the desired end by the same methods that he would ordinarily employ in the treatment of any other case of minor surgical injury. As was to be expected, therefore, the means adopted for the protection of the vaccination wounds have been very various, and different trade firms have undoubtedly reaped an extensive harvest by the introduction and energetic advertisement of special dressings of one and another kind. In Paris, at the time of the

6

writer's visit, a semitransparent material known as "taffetas Marinier," not unlike thin isinglass plaster, and which adheres to the skin when moistened with water, was, he found, invariably employed to protect the vaccinated area during the first few days following the operation, and a somewhat similar substance advertised by an English firm is at present utilized to a considerable extent in England. But during the second week of the process it is essential that some dressing of an absorbent nature should be employed, as it is during this period that oozing from the vesicles occasionally supervenes.

The methods employed for retaining the dressings in position are almost as numerous as the latter themselves. At the Government station, in Lamb's Conduit-street, a dressing composed of a couple of layers of boric lint, kept in place by means of pieces of rubber strapping which do not entirely encircle the arm, is applied at the time of vaccination, and this is replaced by another exactly similar dressing when, a week later, the case returns for inspection of the result. But whatever be the nature of the dressing, the free use beneath it of a dusting powder of boric acid has a most beneficial effect in preventing any undue amount of inflammatory reaction.

Concerning the nature of the instrument best adapted for the purpose of vaccination the author makes a few remarks. Here again each operator will probably attain the greatest measure of success with that instrument to the use of which he has been accustomed. But, in speaking generally, the less complicated it is the better. Again, it is desirable that it should be formed entirely of steel, so that it may be readily sterilized by boiling or by heating to redness in the flame of a spirit lamp, the first method being preferable as not tending to injure the temper of the metal. Possibly the best and certainly the simplest form of instrument is the ordinary triangular-headed surgical needle, which possesses the advantage that on account of its small cost a fresh one, if thought necessary, can be employed for every operation. It is curious to find, on turning to the old literature of smallpox inoculation, how all the processes of vaccination were originally copied from those which the inoculators had gradully elaborated. It was of course to these practitioners that Dr. Jenner was indebted for the model on which he framed his method of arm-to-arm vaccination, and in connection with his choice of an instrument the author was specially interested to find that Dr. Emanuel Timoni, in a paper on the practice of inoculation among the Turks, presented to the Royal Society in 1716 by Dr. Woodward, makes the following statement: "These punctures succeed best in the muscle of the arm. The needle is to be a three-edged surgeon's needle; it may likewise be performed with a lancet."

The manner of operating which affords the most generally successful results consists in blowing out the lymph from the capillary tube in which it is stored on to the surface of the skin at different points, the number and situation of which must correspond with those of the vesicles which it is desired to obtain. The skin, put slightly on the stretch, is then gently scarified, through each droplet of lymph, with the needle or instrument, first in one direction and then in another, more or less at right angles to the first, the drawing of blood being avoided as far as possible. In this way the corium or superficial layer of the skin is thoroughly opened up and in some measure removed, and thus the emulsion is brought into intimate relation with the cells of the true skin beneath. Operating in this fashion and employing lymph of normal potency, it is quite easy to obtain an area of vesiculation satisfying official requirements. Many operators reverse the procedure somewhat, first making their scarification and then rubbing on the lymph. But whether it be that some of the minute scratches are thus closed up, as the lymph is applied, or whatever else the reason, certain it is that the results obtained are usually by no means comparable with those following on the method of scarification through the beads of lymph previously dropped upon the skin.

The operation having been completed, it is well to avoid too great haste in applying the protective dressing, especially if this be of the nature of absorbent pad, or if it be impregnated with some powerful germicide, as in the case of sal-alembroth wool. Some little time necessarily elapses before complete absorption of the vaccine emulsion and the exuded lymph has taken place, and to insure the best results a little exercise of patience is essential.

With the object of hastening this period of drying, the author devised some time ago a method of removing the glycerin from the lymph emulsion after such time as bacteriological tests showed that it had fulfilled its purpose of destroying extraneous microörganisms, and replacing it with an equal amount of an inert fluid of similar specific gravity. But the more rapid drying of the vaccinated area thus obtained appeared hardly to compensate for the extra trouble involved in the special preparation of the lymph.

When every care has been taken to protect the arm during the progress of the vaccination and to prevent the premature detachment of the crusts, the amount of permanent scarring of the skin which remains may be astonishingly slight. This is one of the results of modern methods of vaccination to which as yet attention has hardly been sufficiently directed, although in the future it is likely to prove a matter of considerable importance. There can be little doubt that the huge and deep scars which not infrequently resulted from the vaccination of former years were due to some extent to excessive destruction of skin tissue by microorganisms other than those specific to vaccinia. If this be so, then it becomes apparent that persistence of such large and deep scars, practically throughout life, does not necessarily afford evidence that any equivalent degree of immunity against the infection of smallpox is enjoyed by their possessor.—*Therapeutic Gazette.*

---

### THE TUBERCULOSIS PROBLEM.

The subject of pulmonary tuberculosis is exciting the highest attention of the medical profession as well as many outside of it the world over. Its numerous bearings are being studied by all classes of scientists, for the purpose of solving the various ramifications of its subtle influences.

The third annual session of the American Congress of Tuberculosis will convene in New York June 2d to 4th, when the subject will receive a good overhauling from many points of attack.

The Governors of many States, who are honorary vice-presidents of the Congress, have appointed men of standing as delegates, and a respectable contingent is expected from foreign countries.

There will be, aside from all papers of a miscellaneous nature, four symposiums, arranged each to occupy one session of the body, viz.: "Preventive Legislation, Embracing the Social, Municipal, and State Aspects of Tuberculosis;" "Tuberculosis in Its Pathological and Bacteriological Aspects;" "The Medical and Surgical Aspects of Tuberculosis," and "The Veterinary Aspects of Tuberculosis."

These will each be in charge of a committee who will arrange for the opening papers and for those who will participate.

The great feature of the London Congress last year was its museum, and as we have in our country material for such a museum, it is hoped that the profession will co-operate with the committee in organizing this important department. The catalogue of the museum of the London Congress occupied two hundred pages of printed matter, and embraced drawings, maps, skiographs, photographs, engravings, charts, prints and contributions, besides specimens and illustrations of microscopic and biological work relating in any way to the subject.

It is hoped that the laboratories of the Boards of Health of the cities and States of the Union and the medical colleges and schools will loan specimens of their work to this collection, and that the microscopists and students of biology, pathology and chemistry will enroll and co-operate in this laudable movement to extend the knowledge of the results attained by scientific endeavor in the great work of combating the spread of this great destroyer of mankind, Tuberculosis.

Tuberculosis hospitals for the poor are springing up at many important points, and those able to pay can have the choice of a large number of well-equipped sanatoria, with environment to suit. The Infirmary on Blackwell's Island, established less than three months since, already makes an excellent showing in another column, as to improvement and recovery in a class of patients where little could be expected.

The consensus of opinion seems to favor the sanatorium or the hospital for this class of cases, for many reasons, the chief of which is its communicability.

That pulmonary tuberculosis can be cured in many instances, no physician of experience will question, and as to how it can be done with the greatest number is the all-absorbing question of the hour. The first point is to stop the source of contagion, the second, to fortify the individual against the micro-organism, and we shall succeed in stamping out the dread disease just in proportion as we are able to accomplish these points.

The transmission of the disease by contact is considered now to be infrequent, and the danger of contagion is charged to the dried sputum in the atmosphere, a condition which should be prevented.

As all persons who are exposed to the contagion do not contract the disease, it goes without saying that a congenial soil is necessary to its development. If an antitoxin has been found that will do in this disease what has been done in some others, as is claimed, a great advance has certainly been made in prophylaxis as well as in cure.

It is a curious fact that the discovery of the tubercle bacillus by Koch did not draw any especial attention to the infectious character of the expectoration. Indeed, up to within the last five years this subject was treated with contempt by the medical profession and the public. Now, however, the subject has grown of more importance, until to-day the public have become well impressed with the necessity of care with regard to expectoration. But when it comes to the details of a consumptive, it is somewhat difficult to decide exactly what he ought to do. We cannot demand that he will carry around with him a sputum cup or bottle, or that he shall use paper napkins which he will burn from time to time. In fact, it is quite difficult to disinfect or even to cleanse properly a sputum cup or bottle, and its use is unpleasant both to the patient and those around him. Knopf has devised a flask which can be used by ambulant patients, but which requires the use of both hands, which, naturally attracts attention to him both at home and abroad. Cobb, in a recent number of the *Philadelphia Medical Journal*, has described a cup which can be carried in one's handkerchief, for this purpose. It consist simply of a cup into which is fastened a funnel in such a way that if turned upside down it will not spill, for the major diameter of the flask bears such a relation to the minor diameter that the surface of the liquid will not rise above the mouth of the tube in any position in which the flask may be placed. The patient can conceal this in his handkerchief, and as there is no mechanism to get out of order it can be used easily without attracting attention. The proper way to sterilize it is to simply boil the bottle in an old can. The handkerchief can be folded around the bottle and retained in position by a rubber band around its neck. Cobb states that he has seen patients use it at parties and in the presence of others, and was never able to tell himself whether the patient was using the handkerchief alone or the bottle with it. In this way the patient is able to maintain his self-respect and is not obliged to shock constantly his morbid sensitiveness. It is more convenient than any other form of cup, and, Cobb believes, goes far towards solving the knotty problem of caring for the sputum. It is, of course, necessary that the handkerchief should be sterilized at the same time as the cup. He does not believe in the use of any disinfectants, thinking it is better to depend on boiling alone. In this way it is possible to induce the consumptive in public to use an effective and intelligent method of destroying the tubercle bacilli which he casts off.

The rational treatment of pulmonary tuberculosis demands all the resources at our command, because of its deep-seated and insidious character. It is absolutely necessary that the quality of the atmosphere, the environment, and the rules of hygiene and diet should be thoroughly considered, if we would do our best. The treatment in every respect demands the most rigid individualization. It is useless to attempt to treat every case alike in any respect. Malnutrition being the very foundation of the disease, no effort will avail which does not remove this cause! We should not forget that what is one person's meat is another's poison.

Recovery has been accomplished in some instances with so little difficulty that there is danger that the idea may prevail that it is a simple matter to treat such patients, which is not the case, and harm will ensue if it is so considered.—*Med. Times.*

---

### PNEUMONIA: ITS TREATMENT AND ITS NATURE.

In the course of a discussion on the treatment of pneumonia which is reported in the *Glasgow Medical Journal* for February,

1902, McCall Anderson states that he has no belief whatever in the antiphlogistic treatment (bleeding, blistering, purging, etc.), excepting for certain special reasons ; and if one were to ask him what he considers the very best treatment for pneumonia, he would reply that it is feeding and nursing, and he has no hesitation in saying that if one gets a case of pneumonia in the early stage, and puts the patient under the care of two thoroughly experienced and trained nurses, one for the day and the other for the night time, very often no medicine of any kind is required.

In the treatment of pneumonia there is a great tendency to interfere unduly, and that arises, of course, partly from the fact that in a serious case the friends expect us to treat it energetically. And there is one point—it is of a negative kind of treatment—which he is inclined to insist upon, and it is this: the great importance of not fatiguing and exhausting and distressing the patient by making careful daily, or twice a day, physical examinations of the chest. It is really altogether unnecessary to do so, because one can generally tell quite certainly that a patient is suffering from an acute attack of croupous pneumonia without any examination of the chest at all. There is the rigor at the outset, the decubitus, the flushed cheeks, the herpetic eruption about the lips, the high fever, the tenacious, rusty expectoration, the great perversion in the pulse-respiration ratio—a very important point—and on examination of the urine we will generally find that it is very deficient in chlorides, and often, if there is high fever, contains a little albumin. So when these symptoms, or most of them, are present, we can be absolutely certain of our diagnosis. By this Anderson does not mean that we are not to make any examination, but no unnecessary examination of the chest. We must not treat the patient as if he were laboring under a chronic disease. Then, as regards the minor forms of treatment, some of them do no good, while they are distressing to the patient. The speaker related an incident of which his father once told him, of a patient and friend whom he visited one day, and who said to him, "Weel, doctor, how are you?" "I am not at all well ; I have a very bad cold and I put a mustard poultice on my chest, and it is very sore." "Weel, I'm very glad to hear it, for it will teach you doctors not to order mustard poultices right and left, as if they were nothing."

But if we are to interfere medicinally it must be for the purpose of dealing with some special symptom or complication. Now, for example, there are the two symptoms of severe pain and sleeplessness. If these are not attended to, it might go hard with the patient, and under such circumstances morphine subcutaneously may be used. The only thing that would make one hesitate in the use of morphine would be if the patient had one lung blocked up with pneumonia, while the other was the seat of collateral hyperemia and edema, and the bronchial tubes were choked up with mucus.

There is one point in reference to delirium, in these and other diseases, which must never be overlooked, namely, that in a large proportion of cases the delirium is simply one of the phenomena of the fever. If we put a thermometer into the patient's mouth, in many cases we will find that it registers a very high temperature, and if under these circumstances we resort to the use of antipyretic methods of treatment when the temperature is brought down, the patient often falls into a sound sleep, and on waking the delirium has disappeared. That is a point that should be borne in mind—that delirium is often due to the high fever. Then, as regards the fever itself, it is generally high, but if the case is running its normal course it is a disease of short duration, and therefore—in the case of a person who was previously in good health—it is not such a serious matter, and need not be unduly interfered with. Under these circumstances we need only resort to the milder methods of treatment, such as sponging the patient with vinegar and ice-water, giving him ice to suck, and, if he does not object to it, all his food thoroughly iced. But if there is hyperpyrexia, or if the disease is prolonged, although the fever may not be excessively high—for a prolonged febrile movement is just as dangerous as high temperature for a short time—something more must be done. For instance, we may apply ice-bags to the head, to the affected side of the chest, and in the axillæ, and if we do not succeed in that way in lowering the temperature, we must resort to the use of antipyretics, of whieh the safest is quinine in single doses of ten grains or more if necessary. And in the last resort there is nothing for it but the use of the cold bath. Undoubtedly a great many patients laboring under pneumonia are actually killed, burnt up, by the fever.

As regards the use of stimulants, the author does not put such great faith in them as others seem to do. He thinks that most cases of pneumonia—because it is an exhausting disease—are the better for a little stimulant, particularly in the advancing stages; and as regards their use and the amount to be given, we must be guided by the same principle as would guide us in the use of stimulants in connection with one of the specific fevers. In cases of heart failure, of course, we would have to stimulate the patient freely, and the two medicines which appear to be most useful under these circumstances are subcutaneous injections from time to time of five to seven minims of liquor strychninæ, or perhaps one-thirtieth of a grain of digitalin, or we may combine the two. If there is marked dyspnea and cyanosis, the administration of oxygen mixed with a considerable portion of atmospheric air should certainly be resorted to. The writer, however, does not agree with others in considering it in the light almost of a panacea, nor does he think that the results of oxygen treatment of pneumonia and similar diseases are, on the whole, very satisfactory.

And lastly, as regards the antipneumonic serum, it has not apparently, up to the present time, yielded favorable results; and the speaker concluded with the remark that should he find a patient who resists the methods of treatment which he has endeavored shortly to lay before us, he should certainly be very hopeless as to the result.—*Therapeutic Gazette.*

---

### THE SCIENTIFIC STUDY OF THE CRIMINAL AND DEFECTIVE CLASSES.

It is some years since Lombroso, the eminent Italian, began his studies in criminology and endeavored to trace the impulse of the criminal to an anatomical peculiarity of the brain. Pauline Tarnowsky, the well-known Russian, also contributed to this subject, her principal contribution being a book relating to the anatomical peculiarities of the ears, eyes, etc., of a number of criminal women, including thieves, prostitutes, etc. The first hints thrown out did not fall on fallow ground, and the results so far obtained have been both interesting and satisfactory.

The Fifth International Congress of Criminal Anthropology, in its meeting at Amsterdam, September 9–14, 1901, passed the following resolution:

"The members of the Fifth International Congress of Criminal Anthropology are in favor of the establishment of psycho-physical laboratories for the practical application of physiological psychology to sociological and abnormal or pathological data, especially as found in institutions for the criminal, pauper and defective classes and in hospitals, and also as may be observed in schools and other institutions."

This Congress consists of distinguished specialists from all over Europe, and it is the highest authority. In our country up to date the following associations have passed the same resolution, but referred it to the Department of the Interior: Four National Medical Societies and Associations, the American Medical Association, the Association of American Medical Editors, American Medico-Psychological Associations and the Association for the Study and Cure of Inebriety; thirteen State Medical Societies: Connecticut, Indiana, Kansas, Kentucky, Louisiana, Minnesota, Mississippi Valley Medical Association, North Dakota, New Jersey, Pennsylvania, Texas and Wisconsin; three City Medical Societies: St. Louis, Chicago and Syracuse.

Now that such wide-spread interest has been excited in regard to criminal anthropology, we may expect that the combined studies and records of those who are preparing themselves to enter this field will be rich in results and furnish solutions to many of the intricate and complicated problems of modern sociology. An editorial in the *American Lawyer* of New York gives the best view yet published on the Scientific Study of the Criminal and Defective Classes. It is as follows:

"An effort is being made to establish a laboratory in the Department of the Interior at Washington for the practical application of physiological psychology to sociological and abnormal or pathological data, especially as found in institutions for the criminal, pauper and defective classes and in hospitals, and also as may be observed in schools and other institutions. The defect in our present criminal law is, as we have before remarked, that it regards the crime and not the criminal. It presupposes that all mankind possess an equal power of resistance to anti-social tendencies. It practically lays down as an axiom that the child born of criminal parents, brought up in an environment of crime, is, until he has actually come within the jurisdiction of a magistrate's court, as equally desirable a citizen to all intents and purposes as he who has been reared in the atmosphere of the law-abiding. Until an offense has been committed the law does not recognize the offender. For it the prospective criminal does not exist. Unfortunately, there are some beings who are moral imbeciles. To confine our efforts to punishing crime when committed rather than to preventing its commission, is like the proverbial locking of barn after stealing of horse. Nothing has been done by government as yet to treat the matter scientifically, and when it is considered that $600,000,000 is the annual tribute which, statisticians assure us, society pays to crime, and that the United States has the highest murder rate of any civilized country in the world, one is almost tempted to long for a return to the condition of things when one hundred and sixty offenses were punishable by death, though it be conceded that the death penalty is one of the slightest deterrents to crime." The promoters of the measure have our best wishes. As put by the well-known writer: . . . "The study of man, to be of most utility, must be directed first to the causes of crime, pauperism, alcoholism and other forms of abnormality. To do this the individuals themselves must be studied. As the seeds of evil are usually sown in childhood and youth, it is here that all investigation should commence, for there is little hope of making the world better if we do not seek the causes of social evils at their beginnings."—*The St. Louis Medical and Surgical Journal.*

---

**This Journal and Atlanta Semi-weekly Journal one year for $1.00 cash in advance. No checks. Strictly to new subscribers.**

### THE SO-CALLED "PROUD PEOPLE"; SUBJECTS OF ANAL REFLEX; A PHASE OF HYSTERIA.

BY DR. C. E. WRIGHT, OF INDIANAPOLIS,
Assistant Demonstrator of Anatomy, Medical College of Indiana.

It may be of interest to some of the readers of the *Journal* to hear of a very common disorder about which nothing has ever been recorded in our text-books and with which a goodly number of our profession are unfamiliar. I refer to the so-called "proud people," those individuals who are so highly sensitive to the application of the finger to the anal region that they are thrown into paroxysms over which they have no control and during which they are unaccountable for any actions on their part.

These actions range from those of a very ludicrous nature to those of very grave results either to themselves or others, for they are liable to seize, throw or let fall whatever is nearest or at the time happens to be in their hand. Now laughing, now singing, yelling, whistling, jumping, falling down or what-not at command, they are the source of great amusement to those coming in contact with them and knowing their weakness.

Nor do they value personal safety while thus excited, as is well demonstrated by the case of one man who jumped down a flight of stairs, another who seized a red-hot stovepipe and badly burned himself, while yet another closed the door of an ice-chest on his own head.

The stimulus is not entirely the finger applied, but is accompanied by a chirping noise, as in osculation, although either one or the other, or even a movement as if to carry out the operation, is sufficient to throw these people from a state of reserve into the condition mentioned.

During a paroxysm the subject has a fixed stare and a blank expression, muscular rigidity, the body assuming a certain position, or is thrown into action either immediately or a few seconds after stimulation, perhaps yelling frantically or remaining silent. While another, or perhaps the same individual, when again exposed to this process—maybe, in fact, the next moment—will completely relax, his arms drop, knees bend, in some instances refusing to support the body, the head fall back, having the same fixed stare and blank expression, a constant feature, and again shouting or maintaining silence.

One old fellow that may be mentioned in this class was so moved by whistling that whenever he heard any one whistle he would stop and mark time and was utterly unable to proceed, no matter what the time, place or necessity.

I once saw a young man about twenty years of age who would go wild and beg his tormentors to desist when the finger was pointed at him thirty feet away, though no sound whatever be made.

Some will do or say whatever is uppermost in their minds when excited, carrying out some action or speech suggested by the operator or any one within hearing distance. If, for instance, you ask some question of a subject and then excite him, he will ask the question, unless he has had time to form an answer, when an answer is given instead.

No one who has seen this class of people will doubt the neurotic origin of their disorder, nor doubt their total inability to control themselves when excited, although at times some of them are not influenced by this stimulus for several days and may ultimately recover. They are capable of being molded into various temperaments, made to assume different positions or perform set tasks and repeat certain sentences at the will and pleasure of the operator, although perhaps pleading against doing the same.

They are wont to imitate the tone and style of delivery of the operator in speech or laughter, joining in singing or whistling, often keeping the proper time and striking the proper key.

The negro race is especially prone to this disorder, and in the South are known, so Dr. Alois B. Graham tells me, as the "keechie niggers," some of whom have been known to jump from the river boats into the water while under excitement.

This is an affliction, however, which does not respect rank, race, age or sex, time, place or surroundings, affecting the educated as well as the ignorant.—*Indiana Medical Journal.*

### THE STRANGE ADVENTURES OF AN ANATOMIST'S HEAD.

Xavier Bichat passed a considerable part of his short life in the dead-house, but his own mortal remains appear to have had a more singular fate than usually befalls the fragments of humanity in which he sought so eagerly to discover the secret of life. Writing recently in the *Temps*, M. G. Clarétie says it is well known that when Cuvier was put into his coffin an iron cage was placed over his head so that it might not be stolen as Bichat's had been. A writer in the *Chronique Médicale*, commenting on this statement, says that in 1808 there came in a curious fashion of doing honor to "masters of medicine" by keeping their heads in the condition of anatomical preparations for forty years. Bichat was buried in the St. Catherine Cemetery, in a small corner bought by one of his colleagues at the Hôtel Dieu, and might never have been found if the pious care of friends had not from time to time renewed the marks by which the grave was identified. The cemetery having been closed, Bichat's remains were removed to Père Lachaise. On November 16, 1845, the body was exhumed under the direction of Dr. Denonvilliers, and in the presence of four members of Bichat's family, one of whom was Dr. Adet de Roseville, assistant physician of Saint Lazare, husband of Bichat's niece. The report of the exhumation states that, under a gravestone bearing the inscription, "À Xavier Bichat, par les Membres de la Société d'Instruction Médicale," there was discovered, at a depth of 1 m. 70 cm., in a soil of remarkable dryness, an excellently-preserved skeleton. The cervical vertebræ were perfect, but the head was missing. Further digging failed to bring the head to light. Professor Roux, who was present, came forward and stated that the head of Bichat had "come into his hands" three years after the death of the great anatomist. He described the head, calling attention to the following points: (1) The existence of a fracture of the occipital bone, which he himself had made at the *post-mortem* examination; (2) the obliteration of the alveoli of the first upper molar of the left side and of the corresponding one on the right, which Bichat had had extracted towards the end of his life, after having suffered much from those teeth, as he says himself in his article on the teeth in his *Anatomie Général;* (3) the perfect correspondence of the articular surfaces of the atlas found in the grave with those on the skull. M. Malgaigne had previously arranged in an oak coffin all the bones as they were taken up, and M. Roux completed them by restoring with his own hands the skull which had been so long separated from the skeleton. It may be mentioned that the ceremony of the translation of the relics to Père Lachaise was attended by some 4,000 members of the medical profession.—*Canada New Record.*

---

DIRECT INTRODUCTION OF PURGATIVE INTO THE LARGE INTESTINE IN CASE OF OPERATION FOR SEPTIC PERITONITIS.

A. M. Sheild calls the attention of the profession to a method which he believes is of great utility in the surgery of septic peritonitis—the direct introduction of purgatives into the intestines at the time of operation. It is not too much to say that in many of these cases the patient's life hangs on the possibility of overcoming the paralytic obstruction and the free evacuation of gas and feces. The worse the case the more difficult is this to bring about, since the patient vomits everything he takes by the mouth.

He has hitherto only used this method in cases of perforative appendicitis, and here the performance of the injection is very simple. The nozzle of a small syringe—the hydrocele-injecting syringe is a convenient form—is introduced into the "stump" of the appendix and the solution directly thrown into the cecum. Three drachms of magnesium sulphate, with ten drops of tincture nux vomica, and a drachm of glycerin in an ounce of water is the formula generally employed. Two hours afterwards a turpentine enema is given, and the result has been excellent. He has employed this method in five bad cases of septic peritonitis associated with perforative appendicitis. In every case the results were surprising. And though the number is too small for a pronouncement as to establishing intra-cecal purgatives as a definite line of treatment, yet the cases are sufficiently striking to justify him in urging a trial of it. It is obvious that in other cases the solution could be easily and safely thrown into the colon by means of a hypodermic syringe obliquely introduced. Further evidence may elicit better purgatives than magnesia.—*Brit. Med. Jour.*

